# Evaluation of Biofilm Production and Antibiotic Resistance/Susceptibility Profiles of *Pseudomonas* spp. Isolated from Milk and Dairy Products

**DOI:** 10.3390/foods14071105

**Published:** 2025-03-22

**Authors:** Iván Briega, Sonia Garde, Carmen Sánchez, Eva Rodríguez-Mínguez, Antonia Picon, Marta Ávila

**Affiliations:** Departamento de Tecnología de Alimentos, Instituto Nacional de Investigación y Tecnología Agraria y Alimentaria (INIA), CSIC, Carretera de La Coruña km 7, 28040 Madrid, Spain; ivan.briega@inia.csic.es (I.B.); carmen.sanchez@inia.csic.es (C.S.); minguez@inia.csic.es (E.R.-M.); apicon@inia.csic.es (A.P.)

**Keywords:** *Pseudomonas* spp., spoilage, dairy products, biofilm, antibiotic resistance, phylogeny

## Abstract

Dairy-borne *Pseudomonas* spp., known for causing spoilage, may also exhibit antibiotic resistance and form biofilms, enhancing their persistence in dairy environments and contaminating final products. This study examined biofilm formation and antibiotic resistance in 106 *Pseudomonas* spp. strains isolated from milk, whey, and spoiled dairy products. Phylogenetic analysis (based on partial *ileS* sequences) grouped most strains within the *P. fluorescens* group, clustering into the *P. fluorescens*, *P. gessardii*, *P. koorensis*, and *P. fragi* subgroups. Biofilm formation in polystyrene microplates was assessed at 6 °C and 25 °C by crystal violet staining. After 48 h, 72% and 65% of *Pseudomonas* strains formed biofilms at 6 °C and 25 °C, respectively, with higher biomass production at 6 °C. High biofilm producers included most *P. fluorescens*, *P. shahriarae*, *P. salmasensis*, *P. atacamensis*, *P. gessardii*, *P. koreensis,* and *P. lundensis* strains. The *adnA* gene, associated with biofilm formation, was detected in 60% of the biofilm producers, but was absent in *P. fragi*, *P. lundensis*, *P. weihenstephanensis*, and *P. putida*. Antibiotic susceptibility was tested using the disk diffusion method. All strains were susceptible to amikacin and tobramycin; however, 73% of the strains were resistant to aztreonam, 28% to imipenem and doripenem, 19% to ceftazidime, 13% to meropenem, and 7% to cefepime. A multiple antibiotic resistance index (MARI) > 0.2 was found in 30% of the strains, including multidrug-resistant (*n* = 15) and extensively drug-resistant (*n* = 3) strains. These findings highlight *Pseudomonas* spp. as persistent contaminants and antibiotic resistance reservoirs in dairy environments and products, posing public health risks and economic implications for the dairy industry.

## 1. Introduction

*Pseudomonas* is a diverse genus of aerobic, gram-negative bacilli, motile due to polar flagella [1]. They can grow at a wide temperature range, from 0 °C (e.g., *P. fragi* and *P. fluorescens*) to 41 °C (e.g., the opportunistic pathogen *P. aeruginosa*) [2]. Many species of this genus are able to cause food spoilage in fish, meat, vegetables, and dairy products. *Pseudomonas* is the predominant genus involved in milk and dairy product spoilage, causing taste and visual defects such as milk coagulation and cheese discoloration [3,4,5]. *Pseudomonas* species often dominate during dairy product refrigerated storage [2] and are among the most abundant genera in dairy processing environment core microbiota [6].

Due to its widespread presence in nature, *Pseudomonas* contamination can occur at any point of the dairy chain, and significantly contributes to post-pasteurization contamination [7,8,9]. The ability of certain *Pseudomonas* spp. to form biofilms is a major factor in product contamination, as biofilms are the most frequent contamination source in the dairy industry [8]. Biofilms are microbial communities embedded in extracellular polymeric substances (EPS) secreted by the participating microorganisms. They adhere to biological or abiotic surfaces, protecting the microorganisms from antimicrobials and biocides, while enhancing their resistance to harsh conditions [10,11,12]. Biofilm formation and detachment are regulated by the *quorum sensing* (QS) system [10,13]. In the dairy industry, factors such as humidity, nutrient availability, and raw food cross-contamination promote biofilm development. Specifically, lactose and protein can trigger biofilm formation and QS regulation [14]. *Pseudomonas* can produce EPS in significant amounts and colonize several surfaces [10,13]. Low temperatures and extended storage times favor psychrotrophic *Pseudomonas*, which can form biofilms under diverse conditions, also with other pathogenic species, such as *Listeria monocytogenes* [4]. The goal of controlling biofilm formation would be the prevention of the persistence and spread of spoilage microorganisms, in view of sustainability, reducing food waste by improving the food quality and shelf-life, as well as food safety, and of pathogens across environments, animals, and humans [15]

Antimicrobial resistance (AMR) is a growing global threat to human and animal health, food production, and the environment. AMR makes it increasingly difficult to combat pathogens, especially multidrug-resistant (MDR) microorganisms. It is estimated that bacterial AMR was directly responsible for 1.27 million global deaths in 2019 and contributed to 4.95 million deaths [16]. Non-pathogenic bacterial strains can transfer antibiotic resistance genes to pathogens through a horizontal gene transfer (HGT), both in foods and humans, worsening pathogen eradication from food processing environments, foods, and consumers [2,17]. Moreover, HGT is responsible for spreading antimicrobial resistance within biofilm-forming bacterial communities [18]. Among the environmental AMR reservoirs, the foodborne niche raises special concern for human health, as a significant number of bacteria can reach the human gut through the food chain, where HGT is more likely to occur within the crowded microbial environment of the resident microbiota [17]. The presence of antimicrobial agents along the production chain exerts significant selective pressure, significantly contributing to the emergence of antibiotic-resistant bacteria [19]. Due to their high bacteria and yeast levels, fermented dairy products are among the main sources of foodborne ingested microbes. It becomes crucial to characterize their microbiota in relation to antibiotic resistance. Milk and fresh dairy cheese products represent one of a few “hubs” where commensal or opportunistic pseudomonads frequently cohabit with food microbiota and pathogens, facilitating AMR transmission, and can harbor *Pseudomonas* spp. resistome, which may be transmitted to consumers when ingested [2]. Although MDR in dairy *Pseudomonas* spp. has not emerged as a public health threat yet, several antimicrobial resistance genes have been identified and some dairy isolates have been recognized as MDR [2]. Resistant isolates of *P. lundensis*, *P. fragi*, *P. fluorescens*, and *P. putida* to aztreonam and ciprofloxacin have been reported [20]. Some *P. fluorescens* strains resistant to multiple antibiotics, like aztreonam, trimethoprim–sulfamethoxazole, and carbapenems, were linked to HGT-acquired metallo-β-lactamase genes [21].

Furthermore, although considered non-pathogenic for many years, non-*aeruginosa Pseudomonas* species can cause diseases in immunocompromised patients. Thus, *P. putida*, *P. fluorescens*, *P. stutzeri*, *P. mendocina*, and *P. oryzihabitans* have been related to non-*aeruginosa Pseudomonas* infections [22,23]. A study referring to non-*aeruginosa Pseudomonas* in cystic fibrosis patients identified *P. fluorescens*, *P. putida*, and *P. stutzeri* as the most common species, followed by *P. alcaligenes*, *P. fragi*, *P. mendocina*, *P. nitroreducens*, *P. oleovorans*, *P. oryzihabitans*, and *P. veronii* [24]. *P. putida* was identified as a nosocomial cause of infection, with multi-drug resistance and metallo-β-lactamase production [25]. It can cause severe bacteraemia, pneumonia, and other infections, posing a serious concern in spreading antibiotic-resistant genes to more pathogenic organisms in hospitals [26,27]. Furthermore, the cheese isolate *P. fluorescens* ITEM 17298 decreased the survival probability of infected *Galleria mellonella* larvae, showing moderate pathogenic potential with possible implications in the food safety risk assessment [28]. These findings suggest that non-pathogenic strains could pose direct health risks under specific conditions, which may be exacerbated by resistance to medical treatments.

The aim of this study was to characterize 106 *Pseudomonas* spp. strains isolated from milk and dairy products using a dual approach. First, their biofilm-forming ability was evaluated at refrigeration and ambient temperatures. In addition, their susceptibility/resistance to different classes of antibiotics was assessed. This research helps to evaluate the risk of antibiotic resistance transfer from dairy-borne *Pseudomonas* spp., and their persistence in dairy environments through biofilms. Understanding these risks is crucial to evaluate their impact on consumer health and develop strategies to reduce AMR spread.

## 2. Materials and Methods

### 2.1. Bacterial Strains and Growth Conditions

The 106 dairy-borne *Pseudomonas* strains from the INIA (Instituto Nacional de Investigación y Tecnología Agraria y Alimentaria) culture collection were isolated from Spanish cheese whey, raw and pasteurized milk, and discolored cream and cheeses; they were identified and their spoilage potential characterized [29] (Table S1). *P. fluorescens* ATCC 948 and *P. mosselii* ATCC 49838 (American Type Culture Collection) were used as representative spoilage microorganisms [30,31]. *Pseudomonas* strains were stored at −80 °C in Trypticase Soy Broth with Yeast Extract (TSYE, Oxoid, Basingstoke, UK) and 5% glycerol, and subcultured twice before use. They were grown in Trypticase Soy Broth (TSB, Oxoid), or TSYE agar (TSYEA), and incubated at 25 °C for 24–48 h under aerobic conditions.

### 2.2. Phylogenetic Analysis of Pseudomonas spp.

A phylogenetic analysis of the *ileS* partial sequences [32] from the isolated *Pseudomonas* strains (Table S1) was conducted with the MEGA11 software, v. 11.0.13 [33]. Strains in which either no *ileS* sequence could be obtained or it was too short were not included. The sequences were aligned using the MUSCLE algorithm [34], revised, and adjusted to 630 bp. Gene distances were calculated from nucleotide sequences using the Tamura–Nei method [35], and the phylogenetic tree was generated using the Neighbor-Joining method [36]. A bootstrap analysis was performed with 1000 replications.

The phylogenetic analysis included 32 type or reference *Pseudomonas* strains sequences (accession numbers in Table S2), selected on the basis of BLASTN similarity from a previous study [29], and those of *Pseudomonas* groups reported by Girard et al. [37]: *P. shahriarae* SWRI52T, *P. salmasensis* SWRI126T, *P. putida* NBRC 14164T, *P. psychrophila* CCUG 53877T, *P. poae* LMG 21465T, *P. mosselii* DSM 17497T, *P. lundensis* LMG 13517T, *P. libanensis* DSM 17149T, *P. koreensis* LMG 21318T, *P. grimontii* DSM 17515T, *P. gessardii* LMG 21604T, *P. fulva* NBRC 16637T, *P. fragi* NRRL B-25T, *P. fluorescens* ATCC 13525T, *P. jessenii* DSM 17150T, *P. mediterranea* DSM 16733T, *P. corrugata* LMG 2172T, *P. cedrina* DSM 17516T, *P. canadensis* 2-92T, *P. rhodesiae* DSM 14020T, *P. synxantha* DSM 18928T, *P. orientalis* DSM 17489T, *P. azotoformans* LMG 21611T, *P. atacamensis* M7D1T, *P. aeruginosa* DSM 50071T, *P. tolaasii* NCPPB 2192T, *P. marginalis* DSM 18529T, *P. sivasensis* P7T, *P. veronii* DSM 11331T, *P. fragi* NRRL B-727, *P. saxonica* DSM 108989T, *P. brassicacearum* JCM 11938T, and *P. fragi* NBRC 101046. *Cellvibrio japonicus* Ueda 107 (accession number CP000934.1) was used as the outgroup.

### 2.3. Biofilm Formation by Pseudomonas spp.

The biofilm-forming ability of the 106 isolated *Pseudomonas* spp. strains, and both reference strains, was evaluated at 6 °C and 25 °C, to represent refrigeration and ambient temperatures, respectively. These conditions are commonly encountered during the processing and storage of milk and dairy products. Each strain was inoculated at 1% in TSB, grown for 24 h at 25 °C, and subsequently diluted in fresh TSB to an OD_625nm_ of 0.01. Aliquots (200 µL) were dispensed into wells (8 replicates per strain and temperature) of sterile 96-well polystyrene microplates with lids (Nunc A/S, Roskilde, Denmark), and incubated for 48 h at 6 °C and 25 °C. Un-inoculated TSB served as a negative control. After 48 h, biofilm formation was quantified. Once the liquid was removed, the microplates were washed three times with distilled water. Excess moisture was removed by tapping on paper, followed by air-drying for 30 min. Biofilms were then stained with 250 µL of 0.1% crystal violet solution (Sigma Aldrich, Saint Louis, MO, USA) in distilled water, and incubated at room temperature for 30 min. Then, wells were emptied, washed three times, and allowed to dry. The formed biofilms were solubilized with 250 µL of 30% acetic acid, and after 10 min, the OD_590nm_ was measured using a microplate reader (Multiskan Spectrum, Thermo Electron, Waltham, MA, USA) [38]. The biofilm-forming capacity of the strains was compared using the method described by Stepanović et al. [39]: a cut-off optical density (OD_C_) was defined as three standard deviations above the mean OD of the negative control. Based on OD values, strains were classified into 4 categories: non-producers (NP, OD ≤ ODc), low-ability producers (LP, ODc < OD ≤ 2 × ODc), moderate-ability producers (MP, 2 × ODc < OD ≤ 4 × ODc), and high-ability producers (HP, 4 × ODc < OD).

### 2.4. Molecular Detection of the adnA Gene

To assess the presence of *adnA*, encoding a transcription factor involved in flagellar synthesis and biofilm formation, a 1476 bp fragment of this gene was PCR-amplified using the primers adnA_F (5′-ATGTGGCGTGAAACCAAAAT-3′) and adnA_R (5′-TCAATCATCCGCCTGTTCA−3′) [40]. Whole-cell lysates, prepared by suspending single colonies in 50 µL of sterile deionized water, freezing at −20 °C, and thawing, were used as PCR templates. The PCR mixture contained 0.5 μL of lysate, 2.5 µL of each primer (2 µM), 10 µL DNA AmpliTools Master Mix (Biotools B&M Labs, Madrid, Spain), and deionized water to a final volume of 20 µL. The PCR conditions were 95 °C for 4 min; 30 cycles of 95 °C for 15 s, 60 °C for 30 s, and 72 °C for 90 s; and a final extension at 72 °C for 10 min. PCR products were visualized by agarose gel electrophoresis [29].

### 2.5. Antibiotic Susceptibility/Resistance Profiles

Antimicrobial susceptibility testing was performed following the European Committee on Antimicrobial Susceptibility Testing (EUCAST) guidelines [41], using the disk diffusion method. A selection of 12 antipseudomonal antibiotics (Oxoid) of those recommended by EUCAST and used in human medicine were tested. They represented the following classes: aminoglycosides (amikacin AK, 30 µg; and tobramycin TOB, 10 µg), cephalosporins of third and fourth generation (ceftazidime CAZ, 10 µg; and cefepime FEP, 30 µg), carbapenems (doripenem DOR, 10 µg; imipenem IPM, 10 µg; and meropenem MEM, 10 µg), fluoroquinolones (ciprofloxacin CIP, 5 µg; and levofloxacin LEV, 5 µg), penicillins (piperacillin PRL, 30 µg; and piperacillin–tazobactam TZP, 30–6 µg), and monobactams (aztreonam ATM, 30 µg). Resistance to gentamicin (CN, 10 µg) and trimethoprim–sulfamethoxazole (SXT, 1.25–23.75 µg) was also assessed. *P. aeruginosa* ATCC 27853 served as the reference strain for quality control.

Commercial antibiotic disks were placed on Mueller Hinton (Oxoid) agar plates inoculated with a McFarland 0.5 standard of each *Pseudomonas* strain. Plates were incubated at 30 °C for 20 h, and inhibition zone diameters were measured. Strains were classified as susceptible (S), susceptible to increased exposure (I), or resistant (R) based on EUCAST breakpoints [41]. For gentamicin and trimethoprim–sulfamethoxazole, susceptibility was expressed as the inhibition zone diameter (mm), as no interpretive criteria were available in the EUCAST or Clinical and Laboratory Standards Institute (CLSI) guidelines. Multidrug-resistant (MDR) strains were resistant to at least one antibiotic from three or more classes, while extensively drug-resistant (XDR) strains were resistant to antibiotics from all classes except two or fewer. The Multiple Antibiotic Resistance Index (MARI) was calculated for each strain using 12 antibiotics (excluding gentamicin and trimethoprim–sulfamethoxazole), defined as the ratio of resistant antibiotics to the total tested [42].

## 3. Results and Discussion

### 3.1. Phylogenetic Analysis of Pseudomonas Strains

In a previous study, we identified 106 *Pseudomonas* strains from whey, milk, and spoiled cream and cheese by a BLAST analysis of their *ileS* or *rpoD* sequences [29]. Of the 20 identified species, *P. fluorescens* (19%), *P. fragi* (16%), and *P. lundensis* (12%) were the most prevalent (Tables S1 and S3), but 21% of the strains remained unclassified.

**Figure 1:**
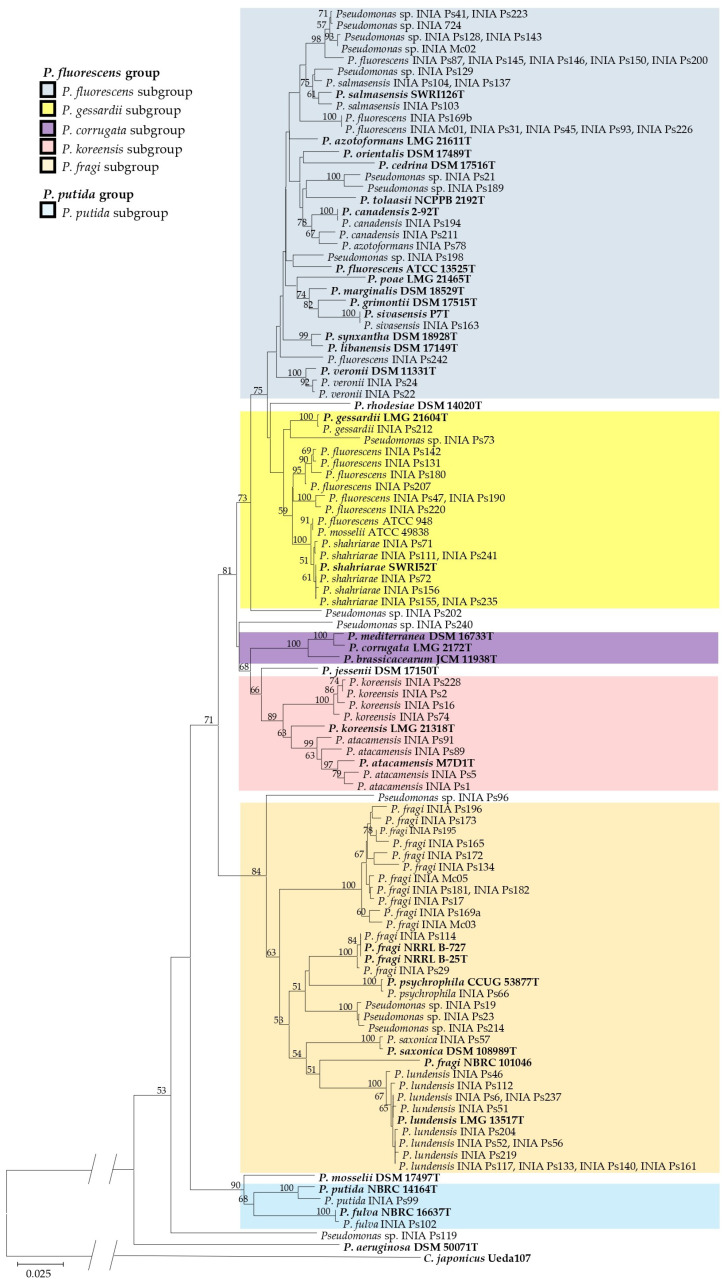
Neighbor-joining phylogenetic tree based on partial *ileS* sequences (630 bp) of selected *Pseudomonas* spp. INIA strains from this study and type/reference *Pseudomonas* strains (in bold). Bootstrap values greater than 50% (from 1000 replications) are indicated at the nodes. Scale bar: 2.5 nt substitutions per 100 nt. *C. japonicus* Ueda107 was used as an outgroup.

In this study, the *ileS* sequences of 95 out of the 106 *Pseudomonas* were phylogenetically affiliated with their closest relatives. The *ileS* gene has been described as a suitable marker for identifying *Pseudomonas* species associated with dairy spoilage [32]. The phylogenetic tree (Figure 1) differentiated *P. fluorescens* and *P. aeruginosa* lineages, with all dairy-borne strains, except *Pseudomonas* sp. INIA Ps119, falling within the *P. fluorescens* one. Within this lineage, two groups were identified: *P. fluorescens* (92 strains) and *P. putida* (2 strains). The *P. fluorescens* group included subgroups such as *P. fluorescens*, *P. gessardii*, *P. koreensis*, and *P. fragi*, while the *P. putida* strains aligned with the *P. putida* group. Four *Pseudomonas* spp. showed a significant evolutionary distance from the rest, suggesting they may represent new species. *Pseudomonas* sp. INIA Ps119 was the most distant from both the *P. fluorescens* and *P. putida* groups, while INIA Ps202, INIA Ps240, and INIA Ps96 formed separate branches within the *P. fluorescens* one. While *ileS* sequencing is useful to assign isolates to taxonomic groups, isolates with highly divergent sequences may require further analysis for precise identification [32].

Thirty-one strains were classified within the *P. fluorescens* subgroup. *P. salmasensis*, *P. canadensis*, *P. sivasensis*, and *P. veronii* grouped with their type strains, while *P. fluorescens* and *P. azotoformans* showed a significant evolutionary distance from their ones. Some *P. fluorescens* strains formed distinct branches, such as INIA Ps87, INIA Ps145, INIA Ps146, INIA Ps150, and INIA Ps200, or INIA Ps242. Others, like INIA Ps169b, INIA Mc01, INIA Ps31, INIA Ps45, INIA Ps93, and INIA Ps226, clustered together but remained distant from the type strain, possibly due to low *ileS* sequence similarity. Additionally, 10 unassigned *Pseudomonas* spp. strains were located within this subgroup. Eighteen strains were classified in the *P. gessardii* subgroup. *P. gessardii* INIA Ps212 and *P. shahriarae* grouped with their type strains, while eight *P. fluorescens* strains were more closely related to *P. gessardii* than *P. fluorescens*. Distinct branches included *P. fluorescens* INIA Ps142, INIA Ps131, INIA Ps180, and INIA Ps207, as well as INIA Ps47, INIA Ps190, and INIA Ps220. *P. fluorescens* ATCC 948 and *P. mosselii* ATCC 49838 formed a separate branch, distant from their type strains. *Pseudomonas* sp. INIA Ps73 also clustered within *P. gessardii*. Additionally, four *P. koreensis* and four *P. atacamensis* strains were classified within the *P. koreensis* subgroup, distant from their type strains.

The *P. fragi* subgroup included 32 strains. *P. fragi* INIA Ps29 and INIA Ps114 grouped with their type strain, while 13 *P. fragi* strains clustered together but remained distant from any reference strain. *P. saxonica* INIA Ps57, *P. psychrophila* INIA Ps66, and 13 *P. lundensis* strains affiliated with their type strains. *Pseudomonas* sp. INIA Ps19, INIA Ps23, and INIA Ps214 formed a distinct branch within the *P. fragi* subgroup.

In the *P. putida* subgroup, *P. putida* INIA Ps99 and *P. fulva* INIA Ps102 grouped with their type strains. The *ileS* sequencing proved to be a fast method for species identification, aligning well with whole-genome sequencing [32].

### 3.2. Biofilm-Forming Ability of Pseudomonas Strains

The ability to form biofilms, associated with dairy product spoilage, was evaluated in TSB at both 6 °C and 25 °C, using crystal violet staining. Based on the OD_590nm_, the strains were classified as: non-producers (NP; OD_590nm_ < 0.249), low-ability producers (LP; 0.249 < OD_590nm_ ≤ 0.498), moderate-ability producers (MP; 0.498 < OD_590nm_ ≤ 0.996), and high-ability producers (HP; OD_590nm_ > 0.996) (Table 1 and Table S3). Biofilm formation and biomass production were strain- and temperature-dependent. After 48 h of incubation, 72% and 65% of the strains formed biofilms at 6 °C and 25 °C, respectively. At 6 °C, 80% of the biofilm-forming strains were classified as HP, 10% as MP, and 10% as LP. At 25 °C, 39% of the biofilm-forming strains were HP, 44% MP, and 17% LP.

Most strains that produced a high biofilm biomass at 25 °C also did at 6 °C, except *P. lundensis* INIA Ps51, INIA Ps56, and INIA Ps117, *P. shahriarae* INIA Ps155, and *P. fluorescens* INIA Ps169b (Table S3). Biofilm production was generally higher at 6 °C, where 30% of the strains produced biofilms with OD_590nm_ ≥ 2 (Table S3), compared to 9% at 25 °C. Cold temperatures enhanced biofilm formation and EPS production in dairy-borne *Pseudomonas* spp. [2,12,38,43,44]. Psychrotrophic *Pseudomonas* can increase EPS secretion in response to cold stress [12,44], which favors bacterial adhesion and biofilm formation through more uniform polysaccharide properties [45].

After incubation at 6 °C, most *P. fluorescens* (17/19) and *P. shahriarae* (5/7) strains, as well as all *P. salmasensis* (3)*, P. canadensis* (2), *P. veronii* (2), *P. koreensis* (4), and *P. atacamensis* (4) strains, and *Pseudomonas* spp. within the *P. fluorescens* (10) and *P. gessardii* (1) phylogenetic subgroups, were classified as high biofilm producers (Table 1). These findings suggest that isolates of these species have significant potential to form biofilms under cold storage and dairy processing conditions. In addition, at 6 °C, 6 out of 13 *P. lundensis* strains, *P. weihenstephanensis* INIA Ps118, *P. sivasensis* INIA Ps163, *P. proteolytica* INIA Ps76, and *Pseudomonas* spp. INIA Ps132, INIA Ps229, and INIA Ps240, produced high biofilm amounts, while *P. fulva* INIA Ps102 and *P. gessardii* INIA Ps212 were moderate producers (Table 1 and Table S3). The highest biomass at 6 °C (OD_590nm_ > 3) was produced by *P. atacamensis* INIA Ps1 and INIA Ps5, *P. koreensis* INIA Ps16, *P. fluorescens* INIA Mc01, *P. lundensis* INIA Ps112, and *P. salmasensis* INIA Ps104 (Table S3).

After incubation at 25 °C, most *P. shahriarae* (6/7) were classified as high biofilm producers, along with strains of *P. fluorescens* (5/19), *P. lundensis* (4/13), *P. koreensis* (2/4), *P. atacamensis* (1/4), and *Pseudomonas* spp. (4/10) within the *P. fluorescens* phylogenetic subgroup, as well as *P. salmasensis* INIA Ps103 (Table 1 and Table S3). Moderate biofilm production at 25 °C was observed for most *P. fluorescens* (12/19) and some *P. lundensis* (3/13), *P. koreensis* (2/4), *P. atacamensis* (2/4), *P. salmasensis* (2/3), *P. veronii* (1/2), *Pseudomonas* spp. (7/16) and *P. solani* INIA Ps105 (Table 1 and Table S3). The highest biomass at 25 °C (OD_590nm_ > 2) was produced by *P. atacamensis* INIA Ps1, *P. lundensis* INIA Ps51 and INIA Ps56, *P. fluorescens* INIA Ps47, *P. koreensis* INIA Ps2, and *P. shahriarae* INIA Ps72 and INIA Ps235 (Table S3).

The reference strain *P. fluorescens* ATCC 948 was classified as LP at 6 °C and MP at 25 °C, in contrast to the wild *P. fluorescens* strains. *P. mosselii* ATCC 49838, most *P. fragi* strains (15/17), *P. azotoformans* INIA Ps78, *P. psychrophila* INIA Ps66, *P. saxonica* INIA Ps57, *P. putida* INIA Ps122, and the *Pseudomonas* spp. strains within the *P. fragi* phylogenetic subgroup, did not form biofilms at any tested temperature (Table 1 and Table S3).

Other studies have also shown that biofilm formation in *Pseudomonas* spp. is a strain-dependent trait that could be related to their surface adaptation. Biofilm formation has been reported in various *Pseudomonas* species, including *P. fluorescens*, *P. lundensis*, *P. libanensis*, *P. koreensis*, *P. azotoformans*, *P. fragi*, *P. putida*, *P. veronii*, *P. gessardii*, *P. psychrophila,* and *P. fulva* [12,14,38,46]. The presence of biofilm-forming *Pseudomonas* in the dairy chain can lead to the failure of biocide treatments, facilitating their spread along with their spoilage enzymes, antibiotic-resistant genes, and foodborne pathogens from mixed biofilms. The observed increase in biomass production at 6 °C is particularly relevant, as dairy products are often stored at refrigerated temperatures during processing and distribution. There is a need for targeted strategies to reduce *Pseudomonas* biofilm formation since conventional cleaning methods are not sufficient. Recent studies have explored the use of ozone, electrolyzed water, organic peroxyacids, ultrasound and lactic acid, low-energy X-ray irradiation, steel coatings, antimicrobial peptides, quorum-sensing inhibitors, bacteriophages, and endolysins, among other strategies, to overcome the persistence of *Pseudomonas* biofilms [2,20,47,48,49,50,51].

### 3.3. Distribution of the adnA Gene in Pseudomonas Strains

Biofilm matrix production is governed by complex regulatory networks that coordinate multiple environmental signals through transcription factors, the second messenger cyclic diguanylate monophosphate (c-di-GMP), and sRNA [52]. The *adnA* gene encodes a *P. fluorescens* transcription factor involved in flagellum synthesis and biofilm formation, homologous to the *P. aeruginosa* and *P. putida fleQ* gene [53,54]. In our study, the *adnA* gene was detected in most biofilm-producing *P. fluorescens* (18/19), *P. salmasensis* (3/3), *P. canadensis* (2/2), *P. shahriarae* (6/6), and *P. koreensis* (2/4) strains, and in the *Pseudomonas* spp. included in the *P. fluorescens* (9/10) phylogenetic subgroup (Table 1 and Table S3). These findings suggest a widespread *adnA* distribution among these species, potentially correlating with biofilm formation. In addition, *adnA* was detected in other biofilm-producing strains such as *P. atacamensis* INIA Ps91 (1/4), *P. sivansensis* INIA Ps163, *P. azotoformans* INIA Ps78, *P. gessardii* INIA Ps212, *P. proteolytica* INIA Ps76, and *Pseudomonas* spp. INIA Ps132, INIA Ps202, INIA Ps229, and INIA Ps240 (Table S3). Our results indicate that *adnA* may not be a specific marker for *P. fluorescens*, as it was detected in other species as well. In line with our findings, other authors have detected *adnA* in most of the tested isolates of the *P. fluorescens* subgroup, including *P. fluorescens*, *P. simiae*, *P. cedrina,* and *P. veronii* [40,46]. The presence of *adnA* alone does not necessarily indicate active biofilm formation under all conditions. Thus, we also detected *adnA* in certain non-biofilm-producing strains, such as *P. fluorescens* INIA Ps180, *P. azotoformans* INIA Ps78, *P. shahriarae* INIA Ps71, and *Pseudomonas* sp. INIA Ps96 and INIA Ps223 (Table S3). However, these strains may have the potential to form biofilms under different conditions or surfaces than those tested in our study.

Some biofilm-producing strains, including those from *P. veronii*, *P. fragi*, *P. lundensis*, *P. weihenstephanensis*, and *P. putida*, lacked the *adnA* gene (Table 1 and Table S3). These results were somewhat expected, since Xu et al. [40] only detected *adnA* in strains of *P. fluorescens,* but not in species like *P. fragi*, *P. lundensis*, *P. putida*, or *P. monteilii.* The findings of Robleto et al. [54] suggest the existence of different biofilm formation pathways depending on the surface, and highlight differences between early and late adhesion events. The *P. fluorescens* strains Pf0-1 and SBW25 produce LapA adhesin and Wss cellulose as key components of their biofilms, respectively [55,56], and may be regulated by c-di-GMP [57]. In *P. fluorescens* UK4 and PF07, the Fap functional amyloid was identified in the biofilms, and RpoN may directly regulate the transcription of *fap* genes, in conjunction with BrfA [58,59,60]. The *aprD* gene has been implicated in regulating the biofilm structure, matrix secretion, and cellular metabolism in *P. fragi* [61]. In *P. putida*, biofilm formation is determined by multiple factors, e.g., LapA and LapF adhesins and the Pea, Peb, cellulose, and alginate polysaccharides, and their transcription is regulated by numerous factors, including FleQ, the sigma factor RpoS, and the DNA-binding protein Fis [57]. Additionally, other *Pseudomonas* extracellular matrix components have been identified through in silico analyses, including the Flp/Tad pilus and poly-N-acetyl-glucosamine [62]. While our study identifies an *adnA* association in some *Pseudomonas* species, transcriptomic or proteomic analyses could further clarify the regulatory networks controlling biofilm development and potential targets for biofilm control in the dairy industry.

### 3.4. Antibiotic Resistance/Susceptibility Profiles in Pseudomonas Strains

The resistance/susceptibility profile of the *Pseudomonas* strains to 14 antibiotics was investigated (Table 2 and Table 3). All strains were susceptible to amikacin and tobramycin. Most strains were classified as susceptible to increased exposure to ciprofloxacin and levofloxacin (99% each), followed by piperacillin and piperacillin–tazobactam (98% each), and cefepime (94%) and ceftazidime (82%). High sensitivity to amikacin, gentamicin, ceftazidime, ciprofloxacin, and imipenem has been reported in cheese isolated *Pseudomonas* spp. [63]. Decimo et al. [64] observed high sensitivity in tank-milk-isolated *Pseudomonas* to fluoroquinolones, aminoglycosides, and piperacillin, and, to a lesser extent, to imipenem, ceftazidime, and cefepime. Furthermore, we observed variable sensitivity to the carbapenem meropenem: 66% of the strains were susceptible, 21% were susceptible to increased exposure, and 13% were resistant, consistent with previous studies [42,64]. The majority of *P. fragi* (82%), *P. lundensis* (69%), *P. weihenstephanensis* (100%), and *Pseudomonas* spp. strains from the *P. fragi* phylogenetic subgroup (67%), as well as *P. fluorescens* ATCC 948 and *P. saxonica* INIA Ps67, were sensitive to all antibiotics (Table 3 and Table S3).

Notably, 73% of the tested *Pseudomonas* strains were resistant to the aztreonam (Table 3 and Table S3), a higher percentage than those reported for other milk-isolated *Pseudomonas* [42,64]. In our study, we also detected strains resistant to imipenem and doripenem (28% each) and, to a lesser extent, to ceftazidime (19%), meropenem (13%), and cefepime (7%) (Table 3 and Table S3). Highly variable resistance percentages have been reported for dairy-borne *Pseudomonas* spp. to these antibiotics [42,63,64]. All of them are β-lactams, belonging to monobactams, carbapenems, or cephalosporins classes, and target enzymes involved in peptidoglycan cross-linking. The main bacterial resistance mechanisms to β-lactams are (i) enzymatic degradation (e.g., β-lactamases encoded by *bla* genes, cephalosporinases, and carbapenemases), (ii) target modification resulting in a lack of β-lactam binding, and (iii) the regulation of β-lactam entry and efflux [65,66]. While *P. aeruginosa* is the most studied *Pseudomonas* species for antibiotic resistance, data on other species are scarce. Meng et al. [42] reported 14 β-lactamase genes linked to β-lactam resistance in milk-isolated *Pseudomonas* spp. No specific monobactam resistance genes have been reported in non-*aeruginosa Pseudomonas* species, though aztreonam resistance in *P. aeruginosa* may result from *ftsI* gene mutations, encoding aztreonam-target FtsI. A homolog (*pbpC*) was found in 10 food-borne *Pseudomonas* spp., but its role in aztreonam resistance remains unclear [67]. Few carbapenem-resistance genes have been identified in non-*aeruginosa Pseudomonas.* The acquisition of exogenous carbapenemase genes, including *bla*_IMP_ and *bla*_VIM_, has been described in *P. putida* [25]. Meropenem resistance in food-borne *Pseudomonas* spp. was mediated by efflux systems, while carbapenem resistance in *P. aeruginosa* resulted from MexAB–OprM efflux pump overexpression and OprD porin loss [68]. Additionally, *P. otitidis* harbored the intrinsic bla_POM_ gene, and *P. putida* exhibited TtgABC efflux system overexpression, also responsible for carbapenem resistance. The carbapenemase bla_PFM-1_ gene was detected in carbapenem-resistant *P. synxantha* [69]. The BIC-1 carbapenemase gene capable of hydrolyzing penicillins, cephalosporins (except ceftazidime), and carbapenems was detected in a *P. fluorescens* from the Seine river [70]. The *bla*_TEM-116_ gene, encoding an extended-spectrum β-lactamase that confers resistance to oxyimino cephalosporins (e.g., cefepime and ceftazidime) and aztreonam, was found in an environmental *P*. *fluorescens* isolate [71].

**Figure 2:**
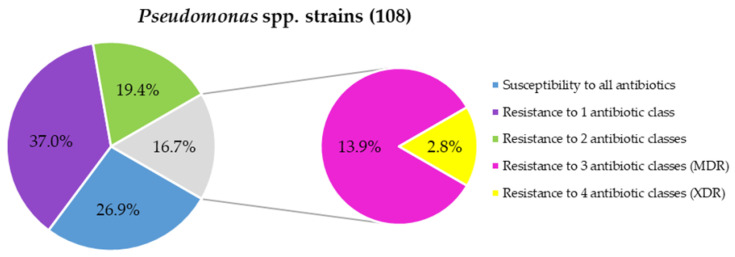
Antibiotic resistance pattern distribution in the studied *Pseudomonas* spp. MDR: multidrug-resistant; XDR: extensively drug-resistant. Strains resistant to 1 antibiotic class: 37% to monobactam. Strains resistant to 2 antibiotic classes: 15.7% to monobactam and carbapenems, and 3.7% to monobactam and cephalosporins. Strains resistant to 3 antibiotic classes: 13.0% to monobactam, cephalosporins, and carbapenems, and 0.9% to monobactam, cephalosporins, and penicillins. Strains resistant to 4 antibiotic classes: 1.9% to monobactam, cephalosporins, fluoroquinolones, and carbapenems, and 0.9% to monobactam, cephalosporins, carbapenems, and penicillins.

The resistance patterns of *Pseudomonas* strains against the 12 antibiotics tested according to EUCAST are illustrated in Figure 2. The majority of *P. fluorescens* (78%) and *P. shahriarae* (100%) strains, as well as 70% of *Pseudomonas* spp. within the *P. fluorescens* phylogenetic subgroup, were resistant to two or more antibiotic classes (Table 3 and Table S3). In total, fifteen *Pseudomonas* strains were resistant to three antibiotic classes, while three additional strains showed resistance to four classes, classifying them as MDR and XDR strains, respectively. Specifically, 85% of *P. shahriarae*, 21% of *P. fluorescens*, and 30% of *Pseudomonas* spp. strains from the *P. fluorescens* phylogenetic subgroup, and *P. mosselii* ATCC 49838 were identified as MDR. Among the XDR strains, *Pseudomonas* sp. INIA Mc02 exhibited resistance to six antibiotics, *P. fluorescens* INIA Ps146 to seven antibiotics, and *P. solani* INIA Ps105 to eight antibiotics. Another study reported that 88.4% of 86 raw milk isolates, representing 11 different *Pseudomonas* species, were MDR [42]. Resistance to β-lactam antibiotics (penicillins, cephalosporins, carbapenems, and monobactams), aminoglycosides, and fluoroquinolones has previously been described in milk and cheese *Pseudomonas* spp. isolates, including *P. fluorescens*, *P. putida*, *P. fulva*, *P. fragi*, *P. lundensis*, *P. mosselii*, *P. libanensis*, *P. gessardii*, and *P. psychrophila*, among others [2,42].

The multiple antibiotic resistance index (MARI) of the tested *Pseudomonas* strains, against the 12 antibiotics recommended by EUCAST, ranged from 0.00 to 0.67, with 30% of strains exhibiting MARI > 0.20, including all MDR and XDR strains (Table S3). A MARI greater than 0.20 indicates a high-risk contamination source where antibiotics are frequently used [72]. In comparison, the MARI values for 86 raw milk *Pseudomonas* isolates, tested against 10 antibiotics, ranged from 0.0 to 0.8, with 59.3% showing a MARI > 0.20 [42]. Additionally, the average MARI for seven raw milk *Pseudomonas* isolates, tested against 14 antibiotics, was 0.36 [72].

There are no EUCAST or CLSI breakpoints for gentamicin and sulfomethoxazole–trimethoprim against *Pseudomonas* spp. However, the FDA interpretative criteria for antibacterial susceptibility testing provide the following breakpoints for *P. aeruginosa* and gentamicin: susceptible ≥ 15 mm; intermediate = 13–14 mm; and resistant ≤ 12 mm [73]. Based on these criteria, the 108 tested *Pseudomonas* spp. strains would be considered susceptible to gentamicin (Table 2), consistent with our observations for the two other aminoglycosides. Regarding sulfomethoxazole–trimethoprim, no inhibition halo was observed against the control *P. aeruginosa* ATCC 27853, since EUCAST notes the intrinsic resistance of *P. aeruginosa* to trimethoprim. However, among the dairy-borne *Pseudomonas* strains, inhibition halos were observed as follows: <7 mm in 7% of strains, 7–20 mm in 64%, and >20 mm in 29% (6 mm antibiotic disk diameter) (Table 2). Knowledge about the intrinsic resistance of non-*aeruginosa Pseudomonas* species is still limited.

Dairy *Pseudomonas* strains with AMR, particularly MDR and XDR, pose a public health risk by potentially transferring antibiotic resistance genes to pathogens, compromising infection treatments in humans and animals. Implementing antimicrobial management programs along the dairy chain could help mitigate AMR by reducing antibiotic use, promoting good hygiene practices and proper veterinary care, and the combination of alternative measures (e.g., probiotics, phytocompounds, and antimicrobial peptides) with modest use of antibiotics [74]. Surveillance systems to monitor the prevalence of antibiotic-resistant strains in dairy products and environments could help in the early identification of resistance patterns and guide interventions. In addition, promising strategies such as plant extracts, antimicrobial peptides, bacteriophages, and physical methods (e.g., high-intensity light pulses, X-rays, ultrasound-steam combinations) have been explored to control *Pseudomonas* and preserve dairy products’ quality [2]. However, further research is needed to develop new and more effective prevention and control methods.

## 4. Conclusions

The results of this study showed that the majority of the *Pseudomonas* strains isolated from Spanish milk and dairy products belonged to the phylogenetic subgroups *P. fluorescens*, *P. gessardii*, *P. koreensis,* and *P. fragi*, all within the *P. fluorescens* group. *Pseudomonas* biofilm production was strongly influenced by temperature, with higher biomass formation under refrigeration. Strains of *P. shahriarae, P. atacamensis*, *P. salmasensis,* and *P. canadensis* were identified as high biofilm producers, alongside well-known dairy *Pseudomonas* such as *P. fluorescens, P. koreensis,* and *P. lundensis.* In contrast, most *P. fragi* and *P. putida* strains exhibited low or no biofilm-forming capacity. Our results highlight the role of the *adnA* gene in biofilm formation in certain *Pseudomonas* species including *P. fluorescens*, *P. shahriarae*, *P. salmasensis,* and *P*. *canadensis*. The tested *Pseudomonas* strains displayed high sensitivity to aminoglycosides and meropenem, and to the increased exposure of fluoroquinolones, penicillins, and cephalosporins. Some species, including *P. fragi*, *P. lundensis*, and *P. weihenstephanensis*, were particularly sensitive to the tested antibiotics. The resistance profiles showed that most *Pseudomonas* strains were resistant to aztreonam and, to a lesser extent, to doripenem and imipenem, and ceftazidime. Multidrug-resistant and extensively drug-resistant strains were mostly identified among *P. fluorescens* and *P. shahriarae*. The presence of highly biofilm-forming, antibiotic-resistant strains within the *P. fluorescens* group presents significant challenges for dairy industry control strategies. These traits may contribute to their persistence in dairy processing equipment and could facilitate the transfer of antibiotic resistance genes to pathogenic bacteria during processing or after dairy product consumption.

## Figures and Tables

**Table 1:** Biofilm-forming abilities of *Pseudomonas* spp. strains based on biomass production after 48 h of incubation in TSB at 6 °C and 25 °C, and *adnA* gene detection.

	Biofilms ^2^ 6 °C, 48 h					Biofilms ^2^ 25 °C, 48 h				*adnA* ^3^
Species ^1^ (*n* = Total no. Strains)	NP	LP	MP	HP		NP	LP	MP	HP	
*P. fluorescens* ATCC 948	0	1	0	0		0	0	1	0	1
*P. fluorescens* (*n* = 19)	1	0	1	17		1	1	12	5	18
*P. salmasensis* (*n* = 3)	0	0	0	3		0	0	2	1	3
*P. veronii* (*n* = 2)	0	0	0	2		0	1	1	0	0
*P. canadensis* (*n* = 2)	0	0	0	2		1	1	0	0	2
*P. azotoformans* (*n* = 1)	1	0	0	0		1	0	0	0	1
*P. sivasensis* (*n* = 1)	0	0	0	1		0	1	0	0	1
* *Pseudomonas* spp. (*n* = 10) ^a^, *P. fluorescens* subgroup	0	0	0	10		1	1	4	4	9
*P. gessardii* (*n* = 1)	0	0	1	0		1	0	0	0	1
*P. shahriarae* (*n* = 7)	1	0	1	5		1	0	0	6	7
*P. proteolytica* (*n* = 1)	0	0	0	1		0	1	0	0	1
* *Pseudomonas* spp. (*n* = 1) ^b^, *P. gessardii* subgroup	0	0	0	1		0	0	0	1	1
*P. koreensis* (*n* = 4)	0	0	0	4		0	0	2	2	2
*P. atacamensis* (*n* = 4)	0	0	0	4		1	0	2	1	1
*P. fragi* (*n* = 17)	15	1	0	1		16	1	0	0	0
*P. lundensis* (*n* = 13)	1	3	2	7		3	2	3	5	0
*P. weihenstephanensis* (*n* = 2)	0	1	0	1		1	1	0	0	0
*P. psychrophila* (*n* = 1)	1	0	0	0		1	0	0	0	0
*P. saxonica* (*n* = 1)	1	0	0	0		1	0	0	0	0
* *Pseudomonas* spp. (*n* = 3) ^c^, *P. fragi* subgroup	3	0	0	0		3	0	0	0	0
*P. putida* (*n* = 2)	2	0	0	0		1	1	0	0	0
*P. fulva* (*n* = 1)	0	0	1	0		1	0	0	0	0
*P. mosselii* ATCC 49838	1	0	0	0		1	0	0	0	0
*P. solani* (*n* = 1)	1	0	0	0		0	0	1	0	0
*Pseudomonas* spp. (*n* = 9) ^d^, other	2	2	2	3		3	1	3	2	5

^1^ Putative identification by BLASTN (NCBI) based on partial sequencing of *ileS* (633 bp) or *rpoD* (736 bp) genes [29]. ^2^ Strains were classified by biofilm biomass production: NP, non-producers (OD_590_ < 0.249); LP, low-ability producers (0.249 < OD_590_ ≤ 0.498); MP, moderate-ability producers (0.498 < OD_590_ ≤ 0.996); HP, high-ability producers (OD_590_ > 0.996). ^3^ *adnA* gene (1436 bp) molecular detection. * *Pseudomonas* strains not identified at the species level were grouped by their phylogenetic subgroup (Figure 1). ^a^ INIA724, INIA Mc02, INIA Ps21, INIA Ps41, INIA Ps128, INIA Ps129, INIA Ps143, INIA Ps189, INIA Ps198, and INIA Ps223; ^b^ INIA Ps73; ^c^ INIA Ps19, INIA Ps23, and INIA Ps214; and ^d^ INIA Ps33, INIA Ps95, INIA Ps96, INIA Ps119, INIA Ps132, INIA Ps188a, INIA Ps202, INIA Ps229, and INIA Ps240.

**Table 2:** Antibiotic susceptibility of *Pseudomonas* strains according to the interpretive criteria established by European Committee on Antimicrobial Susceptibility Testing [41].

Class	Antibiotic	Interpretive Categories ^a^ (Total Strains = 108)		
		S	I	R
Monobactam	Aztreonam	0	29	79
Carbapenems	Doripenem	0	78	30
	Imipenem	0	78	30
	Meropenem	71	23	14
Cephalosporins	Ceftazidime	0	88	20
	Cefepime	0	101	7
Penicillins	Piperacillin	0	106	2
	Piperacillin–tazobactam	0	106	2
Fluoroquinolones	Ciprofloxacin	0	107	1
	Levofloxacin	0	107	1
Aminoglycosides	Amikacin	108	0	0
	Tobramycin	108	0	0
*No breakpoint* ^b^				
Aminoglycosides	Gentamicin	≥15 mm (*n* = 108)		
Folate pathway inhibitors	Sulfamethoxazole–trimethoprim	<7 mm (*n* = 8); 7–20 mm (*n* = 69); >20 mm (*n* = 31)		

^a^ S: susceptible; I: susceptible to increased exposure; R: resistant. ^b^ Susceptibility to gentamicin and sulfamethoxazole–trimethoprim was expressed as the inhibition zone diameter in mm, since no interpretive criteria for these antibiotics were available.

**Table 3:** Antibiotic resistance profiles of *Pseudomonas* strains.

Species ^1^ (Total no. Strains)	Antibiotic Resistance ^2^ (*n* = no. Strains)	Category ^3^
*P. fluorescens* ATCC 948	None (*n* = 1)	
*P. fluorescens* (*n* = 19)	ATM (*n* = 4)	
	ATM, CAZ (*n* = 1)	
	ATM, DOR (*n* = 1)	
	ATM, IPM (*n* = 2)	
	ATM, DOR, IPM (*n* = 4)	
	ATM, DOR, MEM (*n* = 1)	
	ATM, CAZ, IPM (*n* = 1)	MDR
	ATM, DOR, IPM, MEM (*n* = 1)	
	ATM, CAZ, DOR, IPM (*n* = 1)	MDR
	ATM, FEP, DOR, IPM, MEM (*n* = 1)	MDR
	ATM, CAZ, DOR, MEM (*n* = 1)	MDR
	ATM, FEP, CAZ, CIP, DOR, IPM, MEM (*n* = 1)	XDR
*P. salmasensis* (*n* = 3)	ATM (*n* = 2)	
	ATM, DOR, IPM (*n* = 1)	
*P. veronii* (*n* = 2)	ATM (*n* = 2)	
*P. canadensis* (*n* = 2)	ATM (*n* = 2)	
*P. azotoformans* (*n* = 1)	ATM (*n* = 1)	
*P. sivasensis* (*n* = 1)	ATM, CAZ (*n* = 1)	
* *Pseudomonas* spp. (10) ^a^, *P. fluorescens* subgroup	ATM (*n* = 3)	
	ATM, DOR, IPM (*n* = 4)	
	ATM, CAZ, DOR, IPM (*n* = 2)	MDR
	ATM, FEP, CAZ, DOR, IPM, LEV (*n* = 1)	XDR
*P. gessardii* (*n* = 1)	ATM (*n* = 1)	
*P. shahriarae* (*n* = 7)	ATM, DOR, IPM, MEM (*n* = 1)	
	ATM, CAZ, DOR, IPM (*n* = 1)	MDR
	ATM, CAZ, DOR, IPM, MEM (*n* = 4)	MDR
	ATM, FEP, CAZ, DOR, IPM, MEM (*n* = 1)	MDR
*P. proteolytica* (*n* = 1)	ATM, FEP, CAZ, DOR, MEM (*n* = 1)	MDR
* *Pseudomonas* spp. (*n* = 1) ^b^, *P. gessardii* subgroup	ATM, CAZ (*n* = 1)	
*P. koreensis* (*n* = 4)	ATM (*n* = 3)	
	ATM, CAZ (*n* = 1)	
*P. atacamensis* (*n* = 4)	ATM (*n* = 4)	
*P. fragi* (*n* = 17)	None (*n* = 14)	
	ATM (*n* = 3)	
*P. lundensis* (*n* = 13)	None (*n* = 9)	
	ATM (*n* = 4)	
*P. weihenstephanensis* (*n* = 2)	None (*n* = 2)	
*P. psychrophila* (*n* = 1)	ATM (*n* = 1)	
*P. saxonica* (*n* = 1)	None (*n* = 1)	
* *Pseudomonas* spp. (*n* = 3) ^c^, *P. fragi* subgroup	None (*n* = 2)	
	ATM (*n* = 1)	
*P. putida* (*n* = 2)	ATM (*n* = 2)	
*P. fulva* (*n* = 1)	ATM (*n* = 1)	
*P. mosselii* ATCC 49838	ATM, FEP, PRL, TZP (*n* = 1)	MDR
*P. solani* (*n* = 1)	ATM, FEP, CAZ, DOR, IPM, MEM, PRL, TZP (*n* = 1)	XDR
*Pseudomonas* spp. (*n* = 9) ^d^, other	ATM (*n* = 6)	
	ATM, DOR, IPM (*n* = 1)	
	ATM, CAZ, IPM (*n* = 1)	MDR
	ATM, DOR, IPM, MEM (*n* = 1)	

^1^ Putative identification by BLASTN (NCBI) based on partial sequencing of *ileS* (633 bp) or *rpoD* (736 bp) genes [29]. ^2^ CAZ: ceftazidime; FEP: cefepime; DOR: doripenem; IPM: imipenem; MEM: meropenem; CIP: ciprofloxacin; LEV: levofloxacin; PRL: piperacillin; TZP: piperacillin–tazobactam; ATM: aztreonam. ^3^ MDR: multidrug-resistant; XDR: extensively drug-resistant. * *Pseudomonas* strains not identified at the species level are grouped by their phylogenetic subgroup (Figure 1). ^a^ INIA724, INIA Mc02, INIA Ps21, INIA Ps41, INIA Ps128, INIA Ps129, INIA Ps143, INIA Ps189, INIA Ps198, and INIA Ps223; ^b^ INIA Ps73; ^c^ INIA Ps19, INIA Ps23, and INIA Ps214; and ^d^ INIA Ps33, INIA Ps95, INIA Ps96, INIA Ps119, INIA Ps132, INIA Ps188a, INIA Ps202, INIA Ps229, and INIA Ps240.

## Data Availability

The original contributions presented in this study are included in the article/Supplementary Materials. Further inquiries can be directed to the corresponding author.

## Supplementary Materials

The following supporting information can be downloaded at: https://www.mdpi.com/article/10.3390/foods14071105/s1, Table S1: Dairy-borne *Pseudomonas* spp. strains and GenBank accession numbers for their partial *ileS* or *rpoD* sequences; Table S2: Reference and type strains and GenBank accession numbers used for the phylogenetic analysis of partial *ileS* sequences of *Pseudomonas* spp.; Table S3: Biofilm formation, *adnA* gene detection, antibiotic resistance, and multiple antibiotic resistance index (MARI) of dairy-borne *Pseudomonas* spp. in this study.
