# Supplementary material for: Evaluation of Biofilm Production and Antibiotic Resistance/Susceptibility Profiles of Pseudomonas spp. Isolated from Milk and Dairy Products
Source: Foods. 2025 Mar 22;14(7):1105. doi: 10.3390/foods14071105 (PMC11989221; doi:10.3390/foods14071105)
Supplement: Supplementary file 1 [file foods-14-01105-s001.zip › foods-3491726-supplementary.pdf]

Supplementary Material

Evaluation of biofilm production and antibiotic resistance/susceptibility profiles of *Pseudomonas* spp. isolated from milk and dairy products

Iván Briega, Sonia Garde *, Carmen Sánchez, Eva Rodríguez-Mínguez, Antonia Picon and Marta Ávila *

Departamento de Tecnología de Alimentos, Instituto Nacional de Investigación y Tecnología Agraria y Alimentaria (INIA), CSIC, Carretera de La Coruña km 7, 28040 Madrid, Spain; ivan.briega@inia.csic.es (I.B.); carmen.sanchez@inia.csic.es (C.S.); minguez@inia.csic.es (E.R.-M); apicon@inia.csic.es (A.P.)
* Correspondence: sgarde@inia.csic.es (S.G.); arribas@inia.csic.es (M.Á.)

Supplementary material list

Table S1. Dairy-borne *Pseudomonas* spp. strains and GenBank accession numbers for their partial *ileS* or *rpoD* sequences

Table S2. Reference and type strains and GenBank accession numbers used for the phylogenetic analysis of partial *ileS* sequences of *Pseudomonas* spp.

Table S3. Biofilm formation, *adnA* gene detection, antibiotic resistance, and multiple antibiotic resistance index (MARI) of dairy-borne *Pseudomonas* spp. in this study

Table S1. *Pseudomonas* spp. strains from Spanish milk and dairy products and GenBank accession numbers for their partial *ileS* or *rpoD* sequences

Strain	BLAST[1]	Source	*ileS*	*rpoD*
ATCC 49838	*P. mosselii*	Reference strain	PQ157767	—
ATCC 948	*P. fluorescens*	Reference strain	PQ157768	—
INIA 724	*Pseudomonas* sp.	Raw sheep milk	PQ157769	—
INIA Mc01	*P. fluorescens*	Fresh cow milk cheese (Burgos)	PQ157770	—
INIA Mc02	*Pseudomonas* sp.	Fresh cow milk cheese (Burgos)	PQ157771	—
INIA Mc03	*P. fragi*	Fresh cow milk cheese (Burgos)	PQ157772	—
INIA Mc05	*P. fragi*	Fresh cow milk cheese (Burgos)	PQ157773	—
INIA Ps1	*P. atacamensis*	Raw sheep milk	PQ157774	—
INIA Ps2	*P. koreensis*	Raw sheep milk	PQ157775	—
INIA Ps5	*P. atacamensis*	Raw sheep milk	PQ157776	—
INIA Ps6	*P. lundensis*	Raw sheep milk	PQ157777	—
INIA Ps16	*P. koreensis*	Raw sheep milk	PQ157778	—
INIA Ps17	*P. fragi*	Fresh cow milk cheese (Mozzarella)	PQ157779	—
INIA Ps19	*Pseudomonas* sp.	Fresh cow milk cheese (Mozzarella)	PQ157780	—
INIA Ps21	*Pseudomonas* sp.	Fresh cow milk cheese (Mozzarella)	PQ157781	—
INIA Ps22	*P. veronii*	Fresh cow milk cheese (Mozzarella)	PQ157782	—
INIA Ps23	*Pseudomonas* sp.	Fresh cow milk cheese (Mozzarella)	PQ157783	—
INIA Ps24	*P. veronii*	Fresh cow' milk cheese (Mozzarella)	PQ157784	—
INIA Ps29	*P. fragi*	Fresh cow milk cheese (Mozzarella)	PQ157785	—
INIA Ps31	*P. fluorescens*	Fresh cow milk cheese (Mozzarella)	PQ157786	—
INIA Ps33	*Pseudomonas* sp.	Fresh cow milk cheese (Mozzarella)	—	PQ157860
INIA Ps41	*Pseudomonas* sp.	Raw sheep milk	PQ157787	—
INIA Ps45	*P. fluorescens*	Raw goat milk	PQ157788	—
INIA Ps46	*P. lundensis*	Raw goat milk	PQ157789	—
INIA Ps47	*P. fluorescens*	Raw goat milk	PQ157790	—
INIA Ps51	*P. lundensis*	Ripened goat milk cheese (Tronchón)	PQ157791	—
INIA Ps52	*P. lundensis*	Ripened goat milk cheese (Tronchón)	PQ157792	—
INIA Ps53	*P. fragi*	Raw sheep milk	—	PQ157861
INIA Ps56	*P. lundensis*	Ripened goat milk cheese (Tronchón)	PQ157793	—
INIA Ps57	*P. saxonica* sp.	Ripened goat milk cheese (Tronchón)	PQ157794	—
INIA Ps66	*P. psychrophila*	Ripened goat milk cheese (Tronchón)	PQ157795	—
INIA Ps71	*P. shahriarae*	Fresh goat milk cheese	PQ157796	—
INIA Ps72	*P. shahriarae*	Fresh goat milk cheese	PQ157797	—
INIA Ps73	*Pseudomonas* sp.	Fresh goat milk cheese	PQ157798	—
INIA Ps74	*P. koreensis*	Pasteurized sheep milk	PQ157799	—
INIA Ps76	*P. proteolytica*	Pasteurized sheep milk	PQ157800	—

**Strain**	**BLAST**[1]	**Source**	***ileS***	***rpoD***
INIA Ps78	*P. azotoformans*	Pasteurized sheep milk	PQ157801	—
INIA Ps87	*P. fluorescens*	Fresh cow milk cheese	PQ157802	—
INIA Ps89	*P. atacamensis*	Fresh cow milk cheese	PQ157803	—
INIA Ps91	*P. atacamensis*	Raw goat milk	PQ157804	—
INIA Ps93	*P. fluorescens*	Raw goat milk	PQ157805	—
INIA Ps95	*Pseudomonas* sp.	Raw goat milk	PQ157806	—
INIA Ps96	*Pseudomonas* sp.	Raw goat milk	PQ157807	—
INIA Ps99	*P. putida*	Cream	PQ157808	—
INIA Ps102	*P. fulva*	Cream	PQ157809	—
INIA Ps103	*P. salmasensis*	Cream	PQ157810	—
INIA Ps104	*P. salmasensis*	Cream	PQ157811	—
INIA Ps105	*P. solani* sp. nov.	Pasteurized cow milk	—	PQ157862
INIA Ps111	*P. shahriarae*	Raw sheep milk	PQ157812	—
INIA Ps112	*P. lundensis*	Raw sheep milk	PQ157813	—
INIA Ps114	*P. fragi*	Raw sheep milk	PQ157814	—
INIA Ps117	*P. lundensis*	Raw sheep milk	PQ157815	—
INIA Ps118	*P. weihenstephanensis*	Raw sheep milk	—	PQ157863
INIA Ps119	*Pseudomonas* sp.	Cream	PQ157816	—
INIA Ps122	*P. putida*	Cream	—	PQ157864
INIA Ps128	*Pseudomonas* sp.	Raw sheep milk	PQ157817	—
INIA Ps129	*Pseudomonas* sp.	Raw sheep milk	PQ157818	—
INIA Ps131	*P. fluorescens*	Raw sheep milk	—	PQ157865
INIA Ps132	*Pseudomonas* sp.	Raw sheep milk	—	—
INIA Ps133	*P. lundensis*	Raw sheep milk	PQ157819	—
INIA Ps134	*P. fragi*	Raw sheep milk	PQ157820	—
INIA Ps135	*P. weihenstephanensis*	Raw sheep milk	—	PQ157866
INIA Ps137	*P. salmasensis*	Raw sheep milk	PQ157821	—
INIA Ps138	*P. fragi*	Raw sheep milk	—	PQ157867
INIA Ps140	*P. lundensis*	Raw sheep milk	PQ157822	—
INIA Ps142	*P. fluorescens*	Raw sheep milk	PQ561518	—
INIA Ps143	*Pseudomonas* sp.	Tofu	PQ157823	—
INIA Ps145	*P. fluorescens*	Tofu	PQ157824	—
INIA Ps146	*P. fluorescens*	Tofu	PQ157825	—
INIA Ps150	*P. fluorescens*	Tofu	PQ157826	—
INIA Ps155	*P. shahriarae*	Fresh cow milk cheese	PQ157827	—
INIA Ps156	*P. shahriarae*	Fresh cow milk cheese	PQ157828	—
INIA Ps161	*P. lundensis*	Fresh cow milk cheese	PQ157829	—

Strain	BLAST[1]	Source	*ileS*	*rpoD*
INIA Ps163	*P. sivasensis*	Fresh cow milk cheese	PQ157830	—
INIA Ps165	*P. fragi*	Fresh cow milk cheese	PQ157831	—
INIA Ps169a	*P. fragi*	Fresh cow milk cheese	PQ157832	—
INIA Ps169b	*P. fluorescens*	Fresh cow milk cheese	PQ157833	—
INIA Ps172	*P. fragi*	Fresh cow milk cheese	PQ157834	—
INIA Ps173	*P. fragi*	Fresh cow milk cheese	PQ157835	—
INIA Ps180	*P. fluorescens*	Fresh cow milk cheese	PQ561519	—
INIA Ps181	*P. fragi*	Cheese whey	PQ157836	—
INIA Ps182	*P. fragi*	Cheese whey	PQ157837	—
INIA Ps188a	*Pseudomonas* sp.	Cheese whey	—	PQ157870
INIA Ps189	*Pseudomonas* sp.	Cheese whey	PQ157838	—
INIA Ps190	*P. fluorescens*	Cheese whey	PQ157839	—
INIA Ps194	*P. canadensis*	Cheese whey	PQ157840	—
INIA Ps195	*P. fragi*	Raw cow milk	PQ157841	—
INIA Ps196	*P. fragi*	Raw cow milk	PQ157842	—
INIA Ps198	*Pseudomonas* sp.	Raw sheep milk	PQ157843	—
INIA Ps200	*P. fluorescens*	Raw sheep milk	PQ157844	—
INIA Ps202	*Pseudomonas* sp.	Raw goat milk	PQ157845	—
INIA Ps204	*P. lundensis*	Raw goat milk	PQ157846	—
INIA Ps207	*P. fluorescens*	Raw sheep milk	PQ561520	—
INIA Ps211	*P. canadensis*	Raw sheep milk	PQ157847	—
INIA Ps212	*P. gessardii*	Raw sheep milk	PQ157848	—
INIA Ps214	*Pseudomonas* sp.	Fresh sheep milk cheese	PQ157849	—
INIA Ps215	*P. fragi*	Cheese whey	—	PQ157872
INIA Ps223	*Pseudomonas* sp.	Fresh sheep milk cheese	PQ157852	—
INIA Ps219	*P. lundensis*	Fresh sheep milk cheese	PQ157850	—
INIA Ps220	*P. fluorescens*	Fresh sheep milk cheese	PQ157851	—
INIA Ps226	*P. fluorescens*	Fresh sheep milk cheese	PQ157853	—
INIA Ps228	*P. koreensis*	Fresh sheep milk cheese	PQ157854	—
INIA Ps229	*Pseudomonas* sp.	Fresh sheep milk cheese	—	—
INIA Ps235	*P. shahriarae*	Raw sheep milk	PQ157855	—
INIA Ps237	*P. lundensis*	Raw sheep milk	PQ157856	—
INIA Ps240	*Pseudomonas* sp.	Fresh sheep milk cheese	PQ157857	—
INIA Ps241	*P. shahriarae*	Fresh sheep milk cheese	PQ157858	—
INIA Ps242	*P. fluorescens*	Fresh sheep milk cheese	PQ157859	—

[1]Putative identification by BLASTN (NCBI) of the partial sequences of the *ileS* (633 bp) or *rpo*D (736 bp) genes [29].

Table S2. Reference and type strains and GenBank accession numbers used for the phylogenetic analysis of partial *ileS* sequences of *Pseudomonas* spp.

**Strain**	**Accession number**
Pseudomonas veronii DSM 11331T	NZ_JYLL01000033.1
Pseudomonas tolaasii NCPPB 2192T	NZ_CP020369.1
Pseudomonas sivasensis P7T	NZ_JAAOWU010000001.1
Pseudomonas synxantha DSM 18928T	NZ_JYLJ01000016.1
Pseudomonas shahriarae SWRI52T	CP077085.1
Pseudomonas salmasensis SWRI126T	CP077083.1
Pseudomonas rhodesiae DSM 14020T	NZ_VFEU01000008.1
Pseudomonas putida NBRC 14164T	AP013070.1
Pseudomonas psychrophila CCUG 53877T	NZ_VZPR01000001.1
Pseudomonas poae LMG 21465T	NZ_MDFK01000046.1
Pseudomonas orientalis DSM 17489T	NZ_JYLM01000013.1
Pseudomonas mosselii DSM 17497T	CP081966.1
Pseudomonas mediterranea DSM 16733T	LT629790.1
Pseudomonas marginalis DSM 18529T	NZ_VFER01000014.1
Pseudomonas lundensis LMG 13517T	NZ_FNLA01000002.1
Pseudomonas libanensis DSM 17149T	NZ_JYLH01000014.1
Pseudomonas koreensis LMG 21318T	NZ_LT629687.1
Pseudomonas jessenii DSM 17150T	NZ_NIWT01000010.1
Pseudomonas grimontii DSM 17515T	NZ_VFES01000001.1
Pseudomonas gessardii LMG 21604	NZ_FNKR01000003.1
Pseudomonas fulva NBRC 16637T	NZ_BBIQ01000017.1
Pseudomonas fragi NRRL B-727	NZ_LT629783.1
Pseudomonas fragi NBRC 101046	BSSW01000001.1
Pseudomonas fragi NRRL B-25T	NZ_JH604622.1
Pseudomonas fluorescens ATCC 13525T	NZ_LT907842.1
Pseudomonas corrugata LMG 2172T	NZ_LT629798.1
Pseudomonas cedrina DSM 17516T	NZ_MNPW01000004.1
Pseudomonas canadensis 2-92T	NZ_KI628539.1
Pseudomonas brassicacearum JCM 11938T	AP029205.1
Pseudomonas azotoformans LMG 21611T	NZ_LT629702.1
Pseudomonas atacamensis M7D1T	SSBS01000008.1
Pseudomonas aeruginosa DSM 50071T	NZ_JALJXB010000001.1
Pseudomonas saxonica DSM 108989T	VFIO01000001.1

Table S3. Biofilm formation, *adnA* gene detection, antibiotic resistance, and multiple antibiotic resistance index (MARI) of dairy-borne *Pseudomonas* spp. in this study

		Biofilm					Antibiotic resistance		
**Strain**	**BLAST**[1]	**6 °C**[2]		**25 °C**[2]		***adn*A**[3]	**Antibiotics**[4]	**Category**[5]	**MARI**[6]
ATCC 49838	*P. mosselii*	0.01	NP	0.08	NP	-	ATM, FEP, PRL, TZP	MDR	0.33
ATCC 948	*P. fluorescens*	0.28	LP	0.66	MP	+			0.00
INIA 724	*Pseudomonas* sp.	2.48	HP	0.18	NP	+	ATM, DOR, IPM		0.25
INIA Mc01	*P. fluorescens*	3.10	HP	0.81	MP	+	ATM, IPM		0.17
INIA Mc02	*Pseudomonas* sp.	2.93	HP	0.65	MP	+	ATM, FEP, CAZ, DOR, IPM, LEV	XDR	0.50
INIA Mc03	*P. fragi*	2.50	HP	0.39	LP	-			0.00
INIA Mc05	*P. fragi*	0.35	LP	0.24	NP	-			0.00
INIA Ps1	*P. atacamensis*	3.15	HP	3.18	HP	-	ATM		0.08
INIA Ps2	*P. koreensis*	1.72	HP	2.35	HP	-	ATM		0.08
INIA Ps5	*P. atacamensis*	3.13	HP	0.97	MP	-	ATM		0.08
INIA Ps6	*P. lundensis*	2.20	HP	0.06	NP	-			0.00
INIA Ps16	*P. koreensis*	3.12	HP	1.85	HP	+	ATM		0.08
INIA Ps17	*P. fragi*	-0.01	NP	0.02	NP	-			0.00
INIA Ps19	*Pseudomonas* sp.	0.00	NP	0.00	NP	-	ATM		0.08
INIA Ps21	*Pseudomonas* sp.	2.63	HP	1.19	HP	+	ATM, CAZ, DOR, IPM	MDR	0.33
INIA Ps22	*P. veronii*	1.81	HP	0.39	LP	-	ATM		0.08
INIA Ps23	*Pseudomonas* sp.	0.00	NP	0.08	NP	-			0.00
INIA Ps24	*P. veronii*	1.91	HP	0.86	MP	-	ATM		0.08
INIA Ps29	*P. fragi*	-0.01	NP	-0.01	NP	-	ATM		0.08
INIA Ps31	*P. fluorescens*	1.84	HP	0.86	MP	+	ATM, DOR, IPM		0.25
INIA Ps33	*Pseudomonas* sp.	0.28	LP	0.58	MP	-	ATM, DOR, IPM, MEM		0.08
INIA Ps41	*Pseudomonas* sp.	2.30	HP	0.75	MP	+	ATM		0.33
INIA Ps45	*P. fluorescens*	2.36	HP	1.30	HP	+	ATM, IPM		0.17

		Biofilm					Antibiotic resistance		
**Strain**	**BLAST[1]**	**6 °C[2]**		**25 °C[2]**		***adn*A[3]**	**Antibiotics[4]**	**Category[5]**	**MARI[6]**
INIA Ps46	*P. lundensis*	1.51	HP	0.36	LP	-			0.00
INIA Ps47	*P. fluorescens*	2.40	HP	2.54	HP	+	ATM, DOR, MEM		0.25
INIA Ps51	*P. lundensis*	0.28	LP	2.81	HP	-	ATM		0.08
INIA Ps52	*P. lundensis*	1.83	HP	1.52	HP	-			0.00
INIA Ps53	*P. fragi*	0.00	NP	-0.02	NP	-			0.00
INIA Ps56	*P. lundensis*	0.26	LP	2.74	HP	-	ATM		0.08
INIA Ps57	*P. saxonica* sp.	-0.01	NP	-0.01	NP	-			0.00
INIA Ps66	*P. psychrophila*	-0.01	NP	-0.01	NP	-	ATM		0.08
INIA Ps71	*P. shahriarae*	0.12	NP	-0.02	NP	+	ATM, DOR, IPM, MEM		0.33
INIA Ps72	*P. shahriarae*	1.17	HP	2.29	HP	+	ATM, CAZ, DOR, IPM, MEM	MDR	0.42
INIA Ps73	*Pseudomonas* sp.	2.13	HP	1.27	HP	+	ATM, CAZ		0.17
INIA Ps74	*P. koreensis*	1.93	HP	0.63	MP	+	ATM, CAZ		0.17
INIA Ps76	*P. proteolytica*	2.59	HP	0.48	LP	+	ATM, FEP, CAZ, DOR, MEM	MDR	0.42
INIA Ps78	*P. azotoformans*	0.00	NP	-0.02	NP	+	ATM		0.08
INIA Ps87	*P. fluorescens*	1.83	HP	0.67	MP	+	ATM, FEP, DOR, IPM, MEM	MDR	0.42
INIA Ps89	*P. atacamensis*	2.16	HP	0.14	NP	-	ATM		0.08
INIA Ps91	*P. atacamensis*	2.90	HP	0.58	MP	+	ATM		0.08
INIA Ps93	*P. fluorescens*	2.09	HP	0.62	MP	+	ATM, DOR, IPM		0.25
INIA Ps95	*Pseudomonas* sp.	0.68	MP	0.68	MP	-	ATM		0.08
INIA Ps96	*Pseudomonas* sp.	0.08	NP	0.24	NP	+	ATM		0.08
INIA Ps99	*P. putida*	0.20	NP	0.41	LP	-	ATM		0.08
INIA Ps102	*P. fulva*	0.67	MP	0.04	NP	-	ATM		0.08
INIA Ps103	*P. salmasensis*	2.67	HP	1.00	HP	+	ATM, DOR, IPM		0.25
INIA Ps104	*P. salmasensis*	3.02	HP	0.74	MP	+	ATM		0.08

		Biofilm					Antibiotic resistance		
**Strain**	**BLAST**[1]	**6 °C**[2]		**25 °C**[2]		***adn*A**[3]	**Antibiotics**[4]	**Category**[5]	**MARI**[6]
INIA Ps105	*P. solani*	0.01	NP	0.95	MP	-	ATM, FEP, CAZ, DOR, IPM, MEM, PRL, TZP	XDR	0.67
INIA Ps111	*P. shahriarae*	1.31	HP	1.33	HP	+	ATM, FEP, CAZ, DOR, IPM, MEM	MDR	0.50
INIA Ps112	*P. lundensis*	3.03	HP	0.83	MP	-			0.00
INIA Ps114	*P. fragi*	0.00	NP	0.00	NP	-			0.00
INIA Ps117	*P. lundensis*	0.37	LP	1.32	HP	-			0.00
INIA Ps118	*P. weihenstephanensis*	1.23	HP	0.36	LP	-			0.00
INIA Ps119	*Pseudomonas* sp.	0.02	NP	-0.02	NP	-	ATM		0.08
INIA Ps122	*P. putida*	0.13	NP	0.23	NP	-	ATM		0.08
INIA Ps128	*Pseudomonas* sp.	1.37	HP	0.59	MP	+	ATM, DOR, IPM		0.25
INIA Ps129	*Pseudomonas* sp.	1.98	HP	0.62	MP	+	ATM		0.08
INIA Ps131	*P. fluorescens*	1.59	HP	0.75	MP	+	ATM		0.08
INIA Ps132	*Pseudomonas* sp.	1.61	HP	1.38	HP	+	ATM, CAZ, IPM	MDR	0.25
INIA Ps133	*P. lundensis*	1.04	HP	0.16	NP	-	ATM		0.08
INIA Ps134	*P. fragi*	-0.01	NP	0.00	NP	-			0.00
INIA Ps135	*P. weihenstephanensis*	0.46	LP	0.09	NP	-			0.00
INIA Ps137	*P. salmasensis*	2.08	HP	0.68	MP	+	ATM		0.08
INIA Ps138	*P. fragi*	-0.02	NP	0.05	NP	-			0.00
INIA Ps140	*P. lundensis*	2.97	HP	0.25	LP	-			0.00
INIA Ps142	*P. fluorescens*	1.85	HP	1.53	HP	+	ATM, CAZ, DOR, MEM	MDR	0.33
INIA Ps143	*Pseudomonas* sp.	1.57	HP	1.20	HP	+	ATM, DOR, IPM		0.25
INIA Ps145	*P. fluorescens*	1.40	HP	0.58	MP	+	ATM, DOR, IPM		0.25
INIA Ps146	*P. fluorescens*	1.30	HP	0.58	MP	+	ATM, FEP, CAZ, CIP, DOR, IPM, MEM	XDR	0.58
INIA Ps150	*P. fluorescens*	1.64	HP	0.45	LP	+	ATM, DOR, IPM, MEM		0.33
INIA Ps155	*P. shahriarae*	0.86	MP	1.06	HP	+	ATM, CAZ, DOR, IPM, MEM	MDR	0.42

		Biofilm					Antibiotic resistance		
**Strain**	**BLAST**[1]	**6 °C**[2]		**25 °C**[2]		***adn*A**[3]	**Antibiotics**[4]	**Category**[5]	**MARI**[6]
INIA Ps156	*P. shahriarae*	1.89	HP	1.25	HP	+	ATM, CAZ, DOR, IPM	MDR	0.33
INIA Ps161	*P. lundensis*	0.05	NP	0.70	MP	-	ATM		0.08
INIA Ps163	*P. sivasensis*	1.59	HP	0.45	LP	+	ATM, CAZ		0.17
INIA Ps165	*P. fragi*	-0.01	NP	0.03	NP	-			0.00
INIA Ps169a	*P. fragi*	-0.02	NP	0.01	NP	-	ATM		0.08
INIA Ps169b	*P. fluorescens*	0.99	MP	1.37	HP	+	ATM, DOR, IPM		0.25
INIA Ps172	*P. fragi*	0.00	NP	0.19	NP	-			0.00
INIA Ps173	*P. fragi*	0.00	NP	0.01	NP	-	ATM		0.08
INIA Ps180	*P. fluorescens*	0.19	NP	0.04	NP	+	ATM		0.08
INIA Ps181	*P. fragi*	0.00	NP	0.00	NP	-			0.00
INIA Ps182	*P. fragi*	0.00	NP	0.00	NP	-			0.00
INIA Ps188a	*Pseudomonas* sp.	0.29	LP	0.28	LP	-	ATM		0.08
INIA Ps189	*Pseudomonas* sp.	1.46	HP	1.54	HP	+	ATM, CAZ, DOR, IPM	MDR	0.33
INIA Ps190	*P. fluorescens*	1.33	HP	0.75	MP	+	ATM, DOR		0.17
INIA Ps194	*P. canadensis*	1.01	HP	0.00	NP	+	ATM		0.08
INIA Ps195	*P. fragi*	0.00	NP	-0.02	NP	-			0.00
INIA Ps196	*P. fragi*	0.00	NP	-0.01	NP	-			0.00
INIA Ps198	*Pseudomonas* sp.	1.69	HP	1.70	HP	+	ATM		0.08
INIA Ps200	*P. fluorescens*	1.53	HP	0.66	MP	+	ATM, CAZ, DOR, IPM	MDR	0.33
INIA Ps202	*Pseudomonas* sp.	0.90	MP	0.11	NP	+	ATM		0.08
INIA Ps204	*P. lundensis*	2.77	HP	1.13	HP	-			0.00
INIA Ps207	*P. fluorescens*	1.68	HP	0.58	MP	+	ATM, CAZ		0.17
INIA Ps211	*P. canadensis*	1.24	HP	0.46	LP	+	ATM		0.08
INIA Ps212	*P. gessardii*	0.90	MP	0.25	NP	+	ATM		0.08

		Biofilm					Antibiotic resistance		
**Strain**	**BLAST[1]**	**6 °C[2]**		**25 °C[2]**		***adn*A[3]**	**Antibiotics[4]**	**Category[5]**	**MARI[6]**
INIA Ps214	*Pseudomonas* sp.	-0.01	NP	-0.04	NP	-			0.00
INIA Ps215	*P. fragi*	-0.02	NP	-0.02	NP	-			0.00
INIA Ps223	*Pseudomonas* sp.	1.73	HP	0.34	LP	-	ATM, DOR, IPM		0.25
INIA Ps219	*P. lundensis*	0.57	MP	0.10	NP	-			0.00
INIA Ps220	*P. fluorescens*	1.73	HP	1.51	HP	-	ATM		0.08
INIA Ps226	*P. fluorescens*	1.82	HP	0.91	MP	+	ATM		0.08
INIA Ps228	*P. koreensis*	1.87	HP	0.87	MP	-	ATM		0.08
INIA Ps229	*Pseudomonas* sp.	2.47	HP	0.67	MP	+	ATM, DOR, IPM		0.25
INIA Ps235	*P. shahriarae*	2.21	HP	2.20	HP	+	ATM, CAZ, DOR, IPM, MEM	MDR	0.42
INIA Ps237	*P. lundensis*	0.63	MP	0.95	MP	-			0.00
INIA Ps240	*Pseudomonas* sp.	1.04	HP	1.80	HP	+	ATM		0.08
INIA Ps241	*P. shahriarae*	2.70	HP	1.29	HP	+	ATM, CAZ, DOR, IPM, MEM	MDR	0.42
INIA Ps242	*P. fluorescens*	2.22	HP	0.79	MP	+	ATM, CAZ, IPM	MDR	0.25

[1]Putative identification by BLASTN (NCBI) of the partial sequences of the *ileS* (633 bp) or *rpoD* (736 bp) genes [29].
[2]The left column is the $OD_{590\ nm}$. In the right column, strains are classified according to their ability to produce biofilms: NP, non-producers ($OD_{590\ nm} < 0.249$); LP, low-ability producers ($0.249 < OD_{590\ nm} \leq 0.498$); MP, moderate-ability producers ($0.498 < OD_{590\ nm} \leq 0.996$); HP, high-ability producers ($OD_{590\ nm} > 0.996$).
[3]Molecular detection of the *adnA* gene (1476 bp).
[4]CAZ: ceftazidime; FEP: cefepime; DOR: doripenem; IPM: imipenem; MEM: meropenem; CIP: ciprofloxacin; LEV: levofloxacin; PRL: piperacillin; TZP: piperacillin-tazobactam; ATM: aztreonam.
[5]MDR: multi-drug resistant (resistant to at least one antibiotic from 3 or more different antibiotic classes); XDR: extensively drug resistant (resistant to at least one antibiotic of all classes except 2 or less).
[6]MARI: ratio between the number of antibiotics to which the strain is resistant and the total number of antibiotics tested (12).
